# Colonel Thomas Stephenson Weir

**Published:** 1910-04-09

**Authors:** 


Colonel Thomas Stephenson Weir, I.M.S., has died at Bandora, near Bombay. In 1868 he took his L.R.C.S., and his L.M. in the following year. His association with our sanitary administration in India began with his appointment as M.O.H. in 1874 to the Bombay Corporation. The outbreak of bubonic plague in 1896 justified his fears that a panic would be caused by stringent Western methods of disinfection. In 1897 he was appointed Chief Health Officer, which signified the acceptance of his views. He retired in 1905.

